# Everything has changed: the impacts of the COVID-19 pandemic on the transit market in Montréal, Canada

**DOI:** 10.1007/s11116-024-10497-2

**Published:** 2024-05-30

**Authors:** Thiago Carvalho, Ahmed El-Geneidy

**Affiliations:** School of Urban Planning, Faculty of Engineering, McGill University, Montréal, QC Canada

**Keywords:** Transit market; market segmentation, Satisfaction, Telecommuting, Frequency of use

## Abstract

The COVID-19 pandemic has significantly impacted the transit market leading to ridership loss and service cuts. Most of the post-pandemic transit market literature has focused on how to attract those who stopped using transit services, however little attention has been given to how rider profiles have changed. To address this gap, we examine 2019 and 2022 data regarding transit commuters from Montréal, Canada. We apply factor and k-means cluster analyses to derive market segments at both points in time considering satisfaction levels, telecommuting rates, and frequency of transit use. We build upon these analyses to report on overall and mode group-level changes in the transit market. Our market segmentation reveals that captive, captive-by-choice, and choice riders still exist in the current public transit market. However, the share of these groups in the market has changed. The proportion of captive and choice riders has increased while captive-by-choice riders have shrunk in size. Moreover, the post-pandemic market has become mostly composed of infrequent riders and higher rates of telecommuting. We further explore these trends by commute mode (i.e., bus only, metro only, and bus and metro users). The findings from this research can be of interest to practitioners and policymakers as they shed light on the evolution of the perceptions and behaviours of segments of transit riders from before to after pandemic.

## Introduction

The COVID-19 pandemic has adversely impacted the transit market leading to service cuts and decline in ridership (Tirachini and Cats 2020). Transit avoidance behaviours adopted as a prevention measure during the pandemic is likely to have caused a fundamental change in travel preference and behaviour (Mashrur et al. 2023), including increased attractiveness of car ownership (Palm et al. 2022) and increased telecommuting rates (Soria et al. 2023). As transit agencies try to adapt to this new reality, they must find ways to optimize service to avoid a continuous cycle of service cuts and ridership losses. Therefore, it becomes important to understand how transit rider profiles have changed to retain existing users.

Understanding the heterogeneity of travel behavior and preferences is important to derive adaptable and effective strategies and policies aimed at retaining and prospecting transit users (Abenoza et al. 2017; Anable 2005; Bamberg and Schmidt 2003; Donald et al. 2014). For instance, low-income populations have been found to experience low satisfaction levels with transit services (Allen et al. 2020; Fu 2022; Tao et al. 2017; Zhao et al. 2014), which can be attributed to using transit due to a lack of travel options and not due to preference. Conversely, those who take transit out of choice show higher levels of satisfaction (Fu 2022; Zhao et al. 2014). Therefore, transit agencies must investigate the needs and preferences of those captive to transit so that they keep using the network even when other options become available. Occasional users report lower satisfaction levels compared to frequent users and tend to be more concerned with the safety and reliability of transit services (Allen et al. 2019; Hsieh 2023). There is also evidence that user preferences and concerns change based on the mode used (e.g., bus vs. metro) (Maciejewska et al. 2023; Shiftan et al. 2015) due to operational differences.

Even though there is ample evidence on pre-pandemic transit profiles diverging based on socio-demographic, travel, geographic and attitudinal characteristics, there is a lack of research on how profiles have changed in post-pandemic conditions. Researchers have started investigating what service improvements could lure those who stopped using transit services back in the post-pandemic world (Mashrur et al. 2023; Palm et al. 2022; Soria et al. 2023). Mashrur et al. (2023) report that providing more direct lines, improving service reliability, and off-peak discounts are important for those who stopped using transit. Moreover, individuals are placing more value on seamless travel and putting more importance on reducing transfer penalties. Even so, most of the transit segmentation research still reflects old behaviours and preferences indicating a need to update the literature to support transit recovery especially as telecommuting rates have increased.

As with many cities in North America, transit ridership in Montréal, Canada is far from recovered. By the end of 2022, ridership in the region was back to 70% of pre-pandemic levels (STM 2022) signaling a shrinkage of the transit market. In this context, we explore the transit market in the region aiming to answer the following questions (i) How has the transit market changed from pre- (2019) to post-pandemic (2022) conditions? and (ii) How have sub-markets of bus only, metro only, and bus and metro users changed within this timeframe? We analyze these submarkets given known differences in user preferences and behaviors across modes (Maciejewska et al. 2023; van Lierop and El-Geneidy 2016). In the study, we consider changes in travel perceptions and preferences, especially travel satisfaction known to be a main determinant of loyalty to transit services (Machado et al. 2018; Sun et al. 2021; van Lierop et al. 2018), frequency of use, telecommuting rates, and personal characteristics (i.e., income, an important component in identifying captive riders). We apply a combination of factor and k-means clustering analysis to define transit market segments which are used to assess how the transit market has shifted over time.

## Literature review

In this section, we explore the impacts of the COVID-19 pandemic on the transit market and the literature on the segmentation of transit markets.

### The COVID-19 pandemic impacts on the transit market

Transit use changed significantly following the COVID-19 pandemic. Travel restrictions and fear of potential exposure made many give up, even if temporarily, on transit leading ridership to drop up to 90% at the height of the pandemic (DeWeese et al. 2020). Lower fare revenue and reduced service offering were a natural consequence (Parker et al. 2021; Qi et al. 2023) causing a disproportional effect on transit operators and those dependent on transit (Tirachini and Cats 2020; Van Dorn et al. 2020). Under these new conditions, transit agencies had to quickly adapt to plan service for essential workers and those who had no other options to reach essential services (Karner et al. 2023). Meanwhile, many found alternative ways of transport (e.g., active travel and car use) and/or switched to telework and other forms or remote activities (Haider et al. 2023; Rahman et al. 2021; Wilbur et al. 2023). The popularity of telework persists beyond the pandemic (Mohammadi et al. 2023) while transit agencies still struggle to recover pre-pandemic levels of ridership (Tirachini and Cats 2020; Ziedan et al. 2023).

Transit recovery has been slow and challenging. Cuts in service, travel restrictions, telecommuting, and increased rates of remote activities are likely to have caused significant habit disruption leading some transit users to adopt other behaviors, such as car use or cycling (Zhao and Gao 2022), which will likely impact transit mode share in the long term (Karner et al. 2023). Choice riders who were able to stay away from transit during the pandemic due to either having a car available or buying one are less likely to have restarted taking transit (Palm et al. 2022). Another important factor influencing reduction in transit use among this group was the ability to work remotely (Brough et al. 2021; Haider et al. 2023). On the other hand, areas with higher rates of low-income populations and/or people of color, which are more dependent on transit, decreased their use to a lower extent compared to high-income areas (Parker et al. 2021; Qi et al. 2023; Wilbur et al. 2023) and have continued to take transit over time (Brough et al. 2021; Liu et al. 2020; Palm et al. 2022). Disadvantaged populations tended to be overrepresented among transit riders during the early stages of the pandemic (Liu et al. 2020) and to face higher challenges in reaching essential services without transit (Palm et al. 2021) especially as they were more likely to experience services cuts (Kar et al. 2022). Thus, increasing their social vulnerability as their accessibility levels decreased. Those dependent on transit have been found to perceive higher infection risks from transit use compared to choice users, however the influence of risk on behavior was smaller reflecting a “boundness” to keep the behavior (Zhao and Gao 2022).

Despite trends in ridership recovery rates diverging between demographics, those with higher accessibility by transit and higher perceptions of service quality are less likely to have bought a new vehicle and to have intentions to reduce transit use (Palm et al. 2022) reflecting a need for transit agencies to provide service with high quality. Travel satisfaction is a main determinant of a rider’s willingness to use and to recommend transit services, both indicators of loyal behavior (Carvalho et al. 2021; van Lierop et al. 2018). Even so, the service quality attributes valued by riders may differ based on an amalgamation of factors, such as their frequency of use. Hsieh (2023) reports that, in the post-pandemic scenario, those who use transit less frequently value safety and reliability while frequent riders see these attributes as basic and value comfort and customer service more. On this note, metro services, often perceived to have higher service quality by users (Cao et al. 2016), are recovering faster than bus services (Cottreau et al. 2023).

### Transit market segmentation

Traditionally, researchers and practitioners segment the market into two categories: choice and captive riders. “Choice riders” represents a diverse set of transit users who choose transit of their own volition (Guerra 2022; Wilson et al. 1984). These individuals can usually be further segmented into smaller categories based on travel and service preferences, attitudes, and behaviours (Guerra 2022). On the other hand, “captive riders” are classified based on income and lack of alternative travel options, such as access to a car (Beimborn et al. 2003). They tend to be more sensitive to negative experiences and to report lower satisfaction levels (Zhao et al. 2014). More recently, Van Lierop and El-Geneidy (2017) identified a third segment, the captive-by-choice riders. This group is likely to have the financial means to access different modes, however they prefer the experience of taking transit. Other scholars have tried to steer away from this paradigm in assessing transit user heterogeneity. To do so, they use a combination of travel (Allen et al. 2019; Shiftan et al. 2015; Sun and Duan 2019; Tao et al. 2017; Viallard et al. 2019) and personal characteristics (Allen et al. 2020; Fu et al. 2018; Mugion et al. 2018; Tyrinopoulos and Antoniou 2008; Vicente et al. 2020), attitudinal (Cheng et al. 2017; Eldeeb and Mohamed 2020; Fu and Juan 2017; Jamal et al. 2023; Kim and Ulfarsson 2012; Krizek and El-Geneidy 2007; Mesbah et al. 2022; Wang et al. 2022) and geographical (Chen 2016; Grisé and El-Geneidy 2018) variables, and transit use potential (Li et al. 2018).

Nonetheless, even papers published after 2022 still reflect pre-pandemic conditions. As examples, Guerra (2022) uses an amalgamation of data from 2012 to 2015 to understand choice riders. Mesbah et al. (2022) collected face-to-face interviews in Iran over three periods across 2017 and 2018 focusing on perceptions of service quality. Wang et al. (2022) focused on shared-mobility preferences, including fixed-route transit usage, from July to November 2018 in the City of Detroit and Ypsilanti Area. Finally, Jamal et al. (2023) focus on the mode choice attitudes and preferences of millennials and older adults from Hamilton, Canada from October to November 2019. In this sense, we add to the literature by examining both pre-pandemic (2019) and post-pandemic (2022) data sets and comparing the changes in transit rider profiles considering satisfaction levels, telecommuting rates, and frequency of transit use.

## Data

To identify changes in the transit market in Montréal before and after the COVID-19 pandemic, this study draws on the 2019 and 2022 waves of the Montréal Mobility Survey (MMS) collected by the Transportation Research at McGill (TRAM) group. In both waves, the research team employed multiple recruitment strategies to ensure a large and representative sample. As proposed by Dillman et al. (2014), advertisement through a marketing company, social media ads, flyer distribution, and personalized email invitations were implemented. Moreover, the same data-cleaning strategy was employed to both data samples to ensure consistency. The exclusion criteria included a short completion time, incomplete responses, multiple responses from the same email address or IP address, and invalid age or height differences between the waves. Those who placed a pin representing their home, school, and/or work location outside of the Montreal metropolitan area were also excluded. In 2019, 3,520 responses were retained after the cleaning procedures and 4,065 in 2022.

For this study, we focus on a subset of the collected data samples, those who commuted to work by Montréal’s local public transit system (Société de Transport de Montréal). A total of 796 respondents reported commuting to work by public transit in 2019 while 653 had done the same in 2022. For our analyses, we further classify the samples into three groups based on the modes used during their commute trip: those who used only the bus, those who used only the metro, and those who used a combination of both. Those who used commuter trains were excluded from the analysis as this service falls under regional provision. For 2019, this yielded 170 bus users, 304 metro users, and 322 who used both modes. In 2022, the sample contained 144 bus users, 267 metro users, and 242 who used a combination of both. It is important to highlight that a share of individuals answered both waves and are present in the two samples (*N* = 154), however direct comparisons between their responses were not assessed.

The MMS collects a range of information on trip satisfaction, travel preferences, travel behaviour, and personal characteristics. Tables 1 and 2 report the questions assessed from each wave chosen based on the literature on transit markets and the impacts of COVID on transit ridership. Travel satisfaction and travel preference questions were asked using a 5-point Likert scale. The question “how many private automobiles do you have regular access to?” was converted to a dummy variable representing having access to a car (or not). Apart from these questions, we incorporate in our analyses the number of telecommuting days during the workweek, the number of transit trips to work, and household income. We transform the household income question into a dummy variable based on earning less than CAD 60,000 a year to indicate low-income households. Finally, we use the number of transit trips to work to classify respondents’ frequency of transit use. Those commuting to work by transit 4 times a week or more were classified as frequent riders, the remaining were deemed infrequent.

## Methods

### Exploratory factor analyses

Factor analysis identifies the smallest number of single underlying latent constructs, or factors, based on the covariance relationship among the studied variables (Hair et al. 2014). In this sense, we apply this technique to reduce the number of variables to be analyzed while having a minimum loss of information. Given this purpose, we conduct a principal components exploratory factor analysis for each wave and transit mode group (i.e., bus only, metro only, and both) using the psych and factoextra packages in R based on Pearson correlation matrices. The number of factors extracted was defined based on latent root criterion (eigenvalue ≥ 1) and parallel analysis, which has been found to perform better than scree plots in determining the number of components to be retained (Zwick and Velicer 1986). To reduce the likelihood of variables loading highly in more than one factor, varimax was applied as the rotation method (Hair et al. 2014). Only variables with factor loadings greater or equal to 0.450 were retained to ensure significance according to our sample sizes (Hair et al. 2014). Similar levels of explained variance were found among the 2019 and 2022 factor analyses. Factorability of the samples was assessed prior to the analyses by confirming that all variables correlate significantly to at least one other variable (*r* ≥ 0.3), adequate levels of sampling adequacy (KMO ≥ 0.7) and that the observed correlation matrix is not the identity matrix (a significant result for the Bartlett’s Test of Sphericity).

### K-means clustering

K-means clustering is a technique based on a centroid method algorithm. Cluster centroids are recalculated every time a new individual is grouped leading to a new centroid to be derived, which is based on the mean values of the observations in the variables being assessed (Hair et al. 2014). The aim is to minimize the differences within groups while maximizing the differences among them. This technique has been applied several times in the literature and it has been shown to be a good transit segmentation method (Cheng et al. 2017; Dent et al. 2021; Grisé and El-Geneidy 2018; Jacques et al. 2013; Krizek and El-Geneidy 2007; Van Lierop and El-Geneidy 2017; Viallard et al. 2019).

To identify cluster groups in Montréal’s transit market, we use a combination of factor scores, calculated in the previous step (Tables 1 and 2), and independent variables. The independent variables added are safety and cost-related statements for bus only and metro only riders in 2019, cost-related statements for bus only and metro only riders in 2022, the incidence of low-income respondents (dummy variable indicating households earning less than CAD 60,000 per year) and the number of telecommuting days during the workweek for all groups for both waves. Except for household income, all variables were normalized to range from − 1 to 1 to follow a similar distribution pattern as the factor scores. The independent variables are added to either match the variables being considered across all commuter groups or to investigate the effects of income and telecommuting on the market.

As suggested by Damant-Sirois et al. (2014), we tried clustering from three to eight groups. To define the number of clusters, we use transit-specific criteria previously used in the literature as defined by Krizek and El-Geneidy (2007) and replicated by Van Lierop and El-Geneidy (2017). In this sense, we assess the statistical output (cluster characteristics), relevance and transferability to transport policy, previous studies, and common sense and intuition. Complementary, we use silhouette analysis, which can help identify the optimal number of clusters based on the separating distance between them. The final cluster selection is used to analyze mode level and overall changes in the transit market.

## Results

### Exploratory factor analysis

Tables 1 and 2 denote the factors extracted for the 2019 and 2022 data samples. The same set of five factors was found for the group of bus and metro users, namely satisfaction with bus operations, satisfaction with metro operations, perceptions of safety, perceptions of cost, and car access. The obtained factors were later used to identify market segments at both points in time using k-means clustering. Fewer factors were extracted for bus only and metro only users, which is explained by a reduced number of variables available for respondents in those categories. For instance, bus only users did not answer metro-related questions and vice versa. Nonetheless, for these groups, the appropriate variables related to cost and safety in 2019 and cost in 2022 were normalized and incorporated in the clustering to ensure consistency as explained in the methods section.

**Table 1:** Factor loadings for the 2019 sample of transit respondents

Factor	Variable	Bus¹	Metro²	Bus and Metro³
Satisfaction (Bus)	I am satisfied with the length of time I spent on the bus.	0.651	-	0.703
	I felt comfortable when I was on the bus.	0.594	-	0.582
	The waiting time for the bus was reasonable.	0.705	-	0.572
	Overall, I was satisfied with my bus experience during this trip.	0.823	-	0.879
Satisfaction (Metro)	I am satisfied with the length of time I spent on the metro.	-	0.726	0.675
	I felt comfortable when I was on the metro.	-	0.672	0.646
	The waiting time for the metro was reasonable.	-	0.661	0.699
	Overall, I was satisfied with my metro experience during this trip.	-	0.928	0.875
Safety	I felt safe from crime and unwanted attention when I was on the bus.	-	-	0.586
	I felt safe from crime and unwanted attention when I was on the metro.	-	-	0.755
Cost	The cost of taking the bus was reasonable.	-	-	0.771
	The cost of taking the metro was reasonable.	-	-	0.914
Car Access	Being in a neighbourhood where it is practical to move around and park by car (traffic is light, there is good access by car, payment, and availability of parking)	0.868	0.758	0.808
	How many private automobiles do you have regular access to?	0.452	0.738	0.446

¹Variance Explain. (49.7%); KMO (0.720); Bartlett’s Test of Sphericity (χ² = 236.30, d.f. = 15, *p*-value = 0)

²Variance Explain. (56.7%); KMO (0.710); Bartlett’s Test of Sphericity (χ² = 609.36, d.f. = 15, *p*-value = 0)

³Variance Explain. (56.9%); KMO (0.750); Bartlett’s Test of Sphericity (χ² = 1,773.79, d.f. = 120, *p*-value = 0)

**Table 2:** Factor loadings for the 2022 sample of transit respondents

Factor	Variable	Bus¹	Metro²	Bus and Metro³
Satisfaction (Bus)	I am satisfied with the length of time I spent on the bus.	0.689	-	0.737
	I felt comfortable when I was on the bus.	0.617	-	0.607
	The waiting time for the bus was reasonable.	0.521	-	0.596
	Overall, I was satisfied with my bus experience during this trip.	0.939	-	0.864
Satisfaction (Metro)	I am satisfied with the length of time I spent on the metro.	-	0.756	0.738
	The waiting time for the metro was reasonable.	-	0.643	0.586
	Overall, I was satisfied with my metro experience during this trip.	-	0.771	0.814
Safety	I felt safe from crime and unwanted attention when I was on the bus.	0.941	-	0.680
	I felt safe from discrimination and racism when I was on the bus.	0.789	-	0.843
	I felt safe from crime and unwanted attention when I was on the metro.	-	0.708	0.573
	I felt safe from discrimination and racism when I was on the metro.	-	0.747	0.736
Cost	The cost of taking the bus was reasonable.	-	-	0.988
	The cost of taking the metro was reasonable.	-	-	0.624
Car Access	I have access to a car whenever I need it	0.653	0.737	0.839
	How many private automobiles do you have regular access to?	0.938	0.745	0.745

¹Variance Explain. (64.8%); KMO (0.700); Bartlett’s Test of Sphericity (χ² = 465.73, d.f. = 28, *p*-value = 0)

²Variance Explain. (59.5%); KMO (0.730); Bartlett’s Test of Sphericity (χ² = 588.54, d.f. = 21, *p*-value = 0)

³Variance Explain. (64.6%); KMO (0.760); Bartlett’s Test of Sphericity (χ² = 2045.06, d.f. = 136, *p*-value = 0)

### K-means clustering

**Fig. 1:**
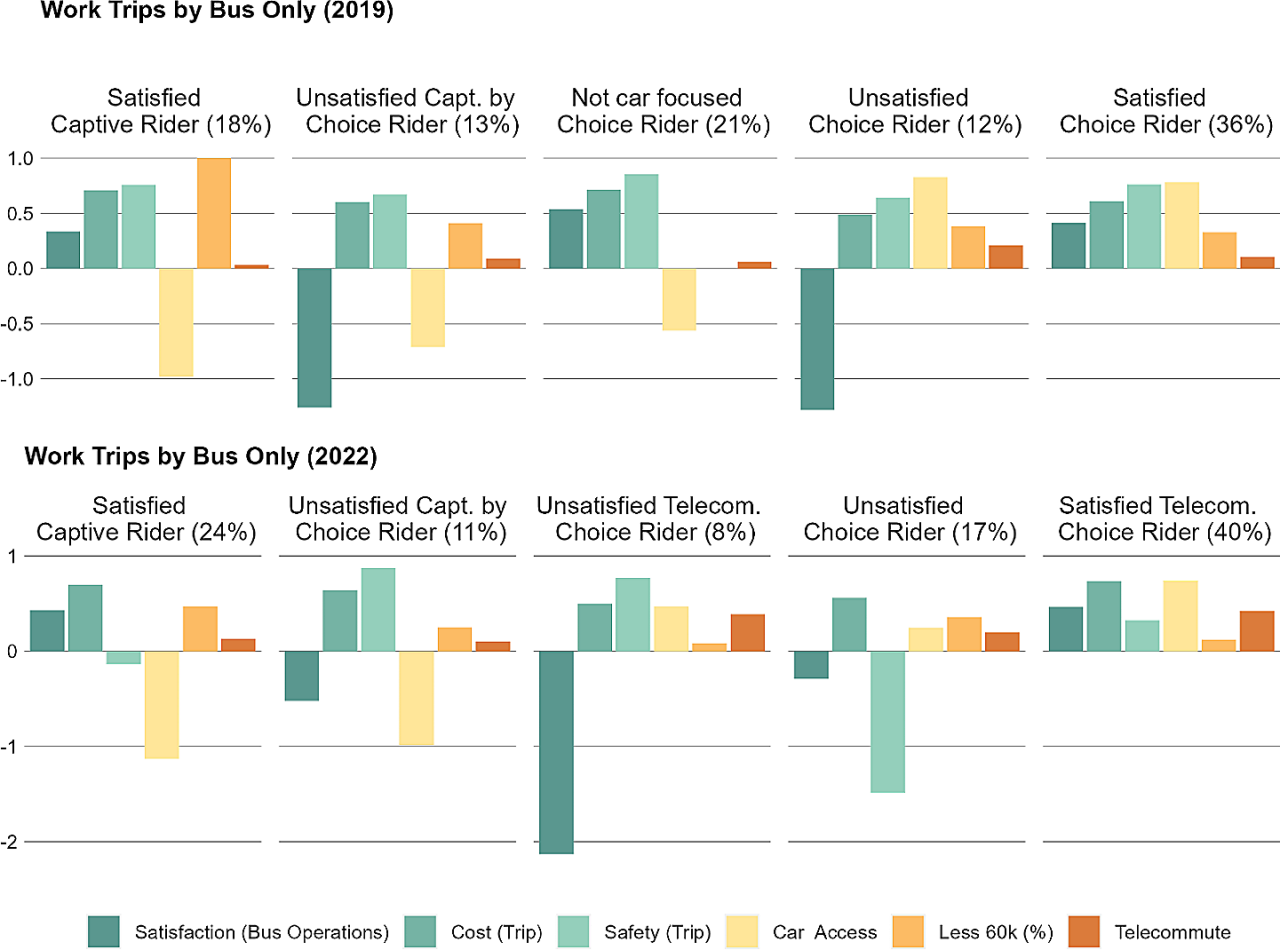
K-means cluster analysis for bus only commuters

**Fig. 2:**
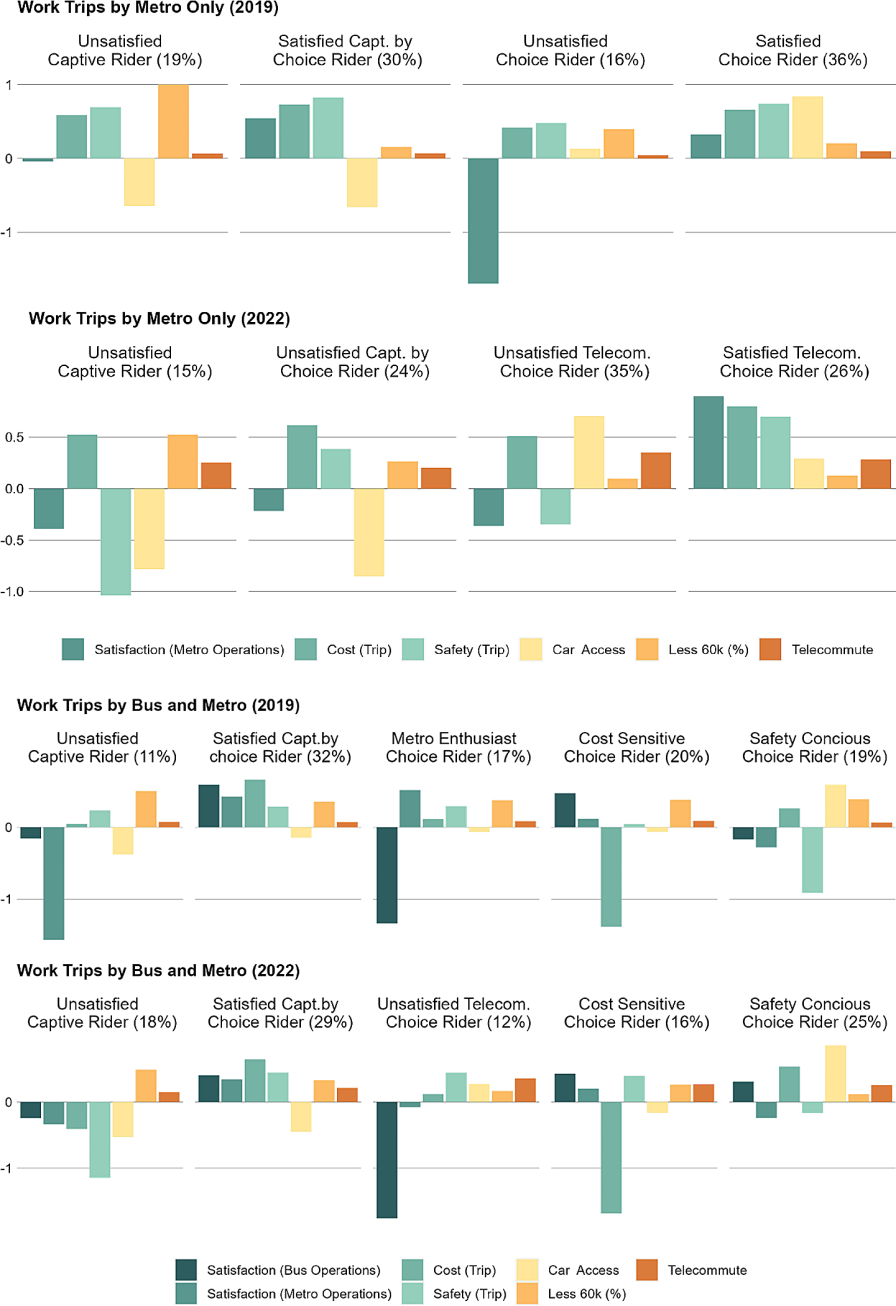
K-means cluster analysis for metro only and bus and metro commuters

Clusters of four or five groups were found to provide the best qualitative description of the market for each mode (Figs. 1 and 2). The selection of clusters is not mode specific and were named based on the prevalence of factors or travellers’ characteristics. For instance, unsatisfied captive riders are found among both metro only and bus and metro users. Nonetheless, several groupings were specific to bus and metro users (e.g., cost sensitive and safety conscious riders). These variances highlight differences in the data structure among different mode groups. A description of the characteristics of each cluster by mode is provided in Table 3, which introduces major discontentment areas for unsatisfied clusters that can serve as basis for policies targeting them.

**Table 3:** Cluster by mode group overtime with reported factors and descriptive variables

Type	Bus Users	Metro Users	Bus and Metro
*Satisfied captive rider*	Do not have access to a car, low income, satisfied with bus service, willing to recommend transit, most do not telecommute, and are frequent transit riders [2019, 2022]	-	-
*Unsatisfied captive rider*	-	Do not have access to a car, low income, unsatisfied with metro service (especially, comfort), willing to recommend transit, most do not telecommute in 2019 but rates increase in 2022, and are frequent transit riders [2019, 2022]	Do not have access to a car, low income, unsatisfied with bus service (especially, travel times, comfort, and waiting times), unsatisfied with metro service (especially, travel times, comfort, and waiting times), not willing to recommend transit, most did not telecommute in 2019 but rates increase in 2022, and are frequent transit riders [2019, 2022]
*Satisfied captive by choice rider*	-	Do not have access to a car, not low income, satisfied with metro service, most do not telecommute, and are frequent transit riders [2019]	Do not have access to a car, not low income, satisfied with bus and metro service, willing to recommend transit, most did not telecommute in 2019 but rates increase in 2022, and are frequent transit riders [2019, 2022]
*Unsatisfied captive by choice rider*	Do not have access to a car, not low income, unsatisfied with bus service (especially, travel time, comfort, waiting times, and cost), willing to recommend transit, most do not telecommute, and are frequent transit riders [2019, 2022]	Do not have access to a car, not low income, unsatisfied with metro service (especially, cost and waiting times), willing to recommend transit, most do not telecommute, and are frequent transit riders [2022]	-
*Not car focused choice rider*	Have access to a car, not low income, does not base their housing decisions based on car infrastructure, satisfied with bus service, most do not telecommute, and are infrequent transit riders [2019]	-	-
*Unsatisfied choice rider*	Have access to a car, not low income, unsatisfied with bus service (especially, travel times, comfort, and waiting times), most do not telecommute, and are frequent transit riders [2019, 2022]	Have access to a car, not low income, unsatisfied with metro service (especially, travel times, comfort, and waiting times), most do not telecommute, and are frequent transit riders [2019]	-
*Satisfied choice rider*	Have access to a car, not low income, satisfied with bus service, most do not telecommute, and are frequent transit riders [2019]	Have access to a car, not low income, satisfied with metro service, most do not telecommute, and are frequent transit riders [2019]	-
*Unsatisfied telecommuter choice rider*	Have access to a car, not low income, unsatisfied with bus service (especially, travel times, comfort, and safety), not willing to recommend transit, most telecommute, and are infrequent transit riders [2022]	Have access to a car, not low income, unsatisfied with metro service (especially, cost, comfort, and safety), willing to recommend transit, most telecommute, and are infrequent transit riders [2022]	Have access to a car, not low income, unsatisfied with bus service (especially, travel times, comfort, and waiting times), satisfied with metro services, not willing to recommend transit, most telecommute, and are infrequent transit riders [2022]
*Satisfied telecommuter choice rider*	Have access to a car, not low income, satisfied with bus service, willing to recommend transit, most telecommute, and are infrequent transit riders [2022]	Have access to a car, not low income, satisfied with metro service, willing to recommend transit, most telecommute, and are infrequent transit riders [2022]	-
*Metro enthusiast choice rider*	-	-	Have access to a car, not low income, unsatisfied with bus service (especially, travel times, comfort, and waiting times), satisfied with metro service, most do not telecommute, and are frequent transit riders [2019]
*Cost sensitive choice rider*	-	-	Have access to a car, not low income, satisfied with bus and metro services, unsatisfied with bus and metro costs, most did not telecommute in 2019 but rates increase in 2022, and are frequent transit riders [2019, 2022]
*Safety conscious choice rider*	-	-	Have access to a car, not low income, unsatisfied with bus and metro operations in 2019 but satisfied in 2022, negative perceptions of safety, most did not telecommute in 2019 but rates increase in 2022, and have become less frequent transit riders [2019, 2022]

2019 = cluster group present in 2019, 2022 = cluster group present in 2022

All cluster groupings were classified as captive, captive-by-choice, and choice users based on income and car access as defined by Krizek and El-Geneidy (2007) and updated by Van Lierop and El-Geneidy (2017). Consequently, those with low income and no car access are categorized as captive users. Choice users are those who have car access and captive-by-choice users are those who are not categorized as having low income and have no car access. This classification is used to identify and characterize the changes in the transit market in the following sections.

The analysis above reveals that captive, captive by choice and choice riders still exist in the current public transit market, which is consistent with previous research, whilst the percentages of these groups in the market has differed as well as the frequency of usage among each group. In the following section, changes in the market at the overall and mode group level are examined.

## Changes in the transit market

**Fig. 3:**
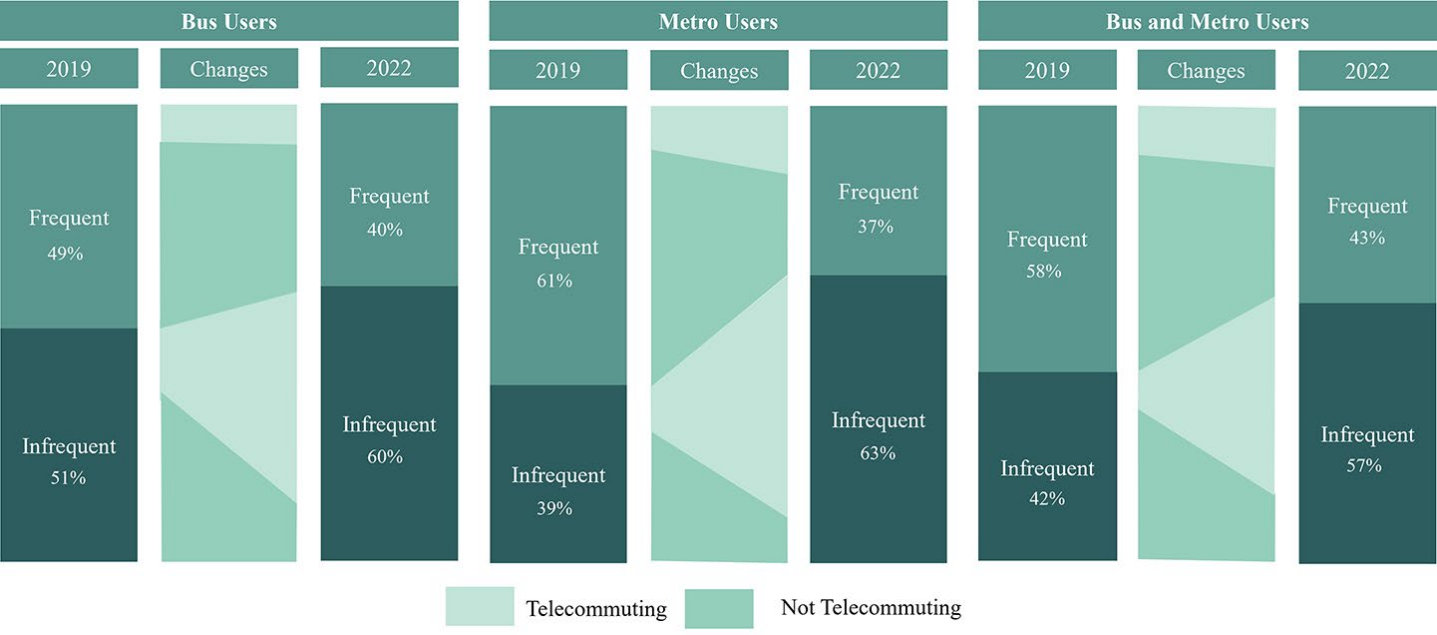
Changes in frequency of use and telecommuting rates by mode group

Transit commuters have become significantly more infrequent after the pandemic (χ² = 42.96, *p* < 0.001). In 2019, 57% of our sample commuted to work by transit at least four times a week (frequent users). After the pandemic, the share of frequent users was reduced by 17%. One explanation for the decrease in frequency of use is the growth in telecommuting. In 2019, infrequent riders telecommuted, on average, 0.5 (s.d. = 1.1) days per week increasing to 2.6 (s.d. = 1.7) in 2022. In comparison, frequent users telecommuted 0.3 (s.d. = 0.7) days on average in 2019 and 0.6 (s.d. = 1.2) in 2022. Both changes are significant at a 0.001 level. Overall, 21% of respondents telecommuted at least one day a week in 2019 while 62% did so in 2022. These trends reflect fundamental changes in how transit systems are being used and how they should be designed. Figure 3 depicts the existence of similar patterns of decrease in frequency of use and increase in telecommuting rates, especially among infrequent users, across all mode groups.

Considering the transit market shares of captive, captive by choice, and choice users found in Sect. 5.2, Table 4 reports on the changes in shares, average number of telecommuting days per week, and average number of transit trips to work per week by mode group. Table 5 displays changes in overall satisfaction towards metro and bus service by mode group. Trends at the overall and mode group level are further explored in the following subsections. A summary of the changes can be found in Table 6.

**Table 4:** Changes in market shares, telecommuting, and transit trips to work within transit market segments by mode group over time

Segments	Bus Users			Metro Users			Bus and Metro Users		
	2019	2022	Diff.	2019	2022	Diff.	2019	2022	Diff.
***Captive***	**18%**	**24%**	**6%**	**19%**	**15%**	**-4%**	**11%**	**18%**	**7%**
Telecommute¹	0.2 (0.5)	0.8 (1.5)		0.3 (0.9)	1.8 (1.6)	***	0.4 (1.0)	1.0 (1.3)	*
Trips to Work²	3.00 (2.3)	4.5 (3.1)		4.0 (2.1)	2.7 (2.1)	*	4.3 (3.0)	4.3 (2.2)	*
***Captive by choice***	**13%**	**11%**	**-2%**	**30%**	**24%**	**-6%**	**32%**	**29%**	**-3%**
Telecommute¹	0.5 (1.3)	0.6 (0.9)		0.3 (0.8)	1.4 (1.7)	***	0.3 (0.8)	1.5 (1.9)	***
Trips to Work²	3.6 (3.5)	3.9 (1.4)		4.0 (2.1)	3.4 (2.0)	***	3.5 (2.0)	3.6 (2.2)	
***Choice***	**69%**	**65%**	**-4%**	**51%**	**61%**	**10%**	**57%**	**53%**	**-4%**
Telecommute¹	0.5 (1.1)	2.2 (1.8)	***	0.4 (0.9)	2.2 (1.7)	***	0.4 (1.0)	2.0 (1.8)	***
Trips to Work²	3.0 (2.2)	2.7 (2.1)	***	3.5 (2.6)	2.9 (2.1)	***	3.6 (2.7)	2.9 (2.2)	***

^1^Avg. number of telecommuting days per week; ²Number of transit trips to work per week; ³Significance of Pearson Chi-Square Test: * *p* < 0.05 ** *p* < 0.01 *** *p* < 0.001.

**Table 5:** Changes in satisfaction within transit market segments by mode group overtime

Mode	Cluster	Year	Bus Satisfaction (Mean)	Diff.¹	Metro Satisfaction (Mean)	Diff.¹
Bus only	Captive	2019	4.3 (0.4)		-	
	Captive	2022	4.3 (0.6)		-	
	Captive by choice	2019	2.7 (0.7)	*	-	
	Captive by choice	2022	3.4 (0.6)		-	
	Choice	2019	3.9 (0.7)		-	
	Choice	2022	3.8 (1.0)		-	
Metro only	Captive	2019	-		3.9 (0.3)	*
	Captive	2022	-		3.6 (0.7)	
	Captive by choice	2019	-		4.4 (0.5)	***
	Captive by choice	2022	-		4.1 (0.4)	
	Choice	2019	-		3.6 (0.9)	***
	Choice	2022	-		4.3 (0.7)	
Bus and Metro	Captive	2019	3.1 (1.0)		2.3 (0.7)	***
	Captive	2022	3.2 (0.9)		3.5 (0.8)	
	Captive by choice	2019	4.1 (0.5)		4.3 (0.5)	*
	Captive by choice	2022	4.2 (0.5)		4.5 (0.6)	
	Choice	2019	3.2 (1.0)	***	3.8 (0.6)	***
	Choice	2022	3.5 (1.1)		4.1 (0.6)	

^1^Significance of Pearson Chi-Square Test: * *p* < 0.05 ** *p* < 0.01 *** *p* < 0.001

### Captive riders

From 2019 to 2022, the share of captive riders has marginally increased (2019 = 16%, 2022 = 18%, + 2%). The increase is most noticeable within the bus only (+ 6%) and bus and metro (+ 7%) mode groups. This trend reflects a loss in choice users who are likely commuting by other means. The metro only portion of the sample was the only group to have a reduction in captive users (-4%). At both points in time, captive riders tend to have higher shares of frequent riders (2019 = 61%, 2022 = 49%) and non-telecommuters (2019 = 84%, 2022 = 52%) when compared to captive-by-choice and choice riders.

Even though shares of telecommuters and infrequent riders have increased across all mode groups, most captive bus riders do not telecommute and are frequent riders. People in this group have the lowest telecommuting rates across all modes and market segments both before and after the pandemic as reported in Table 4. Similarly, bus and metro users tend to not telecommute and to be frequent transit riders. Conversely, 70% of captive metro users telecommute at least once a week and they tend to make less trips to work on average.

Bus only captive riders have remained satisfied with bus services in the Montréal Island. At both points in time, most agree to be satisfied with travel and waiting times, to feel comfortable while on the bus, to feel that the cost of taking the bus is reasonable, and to feel safe from crime and unwanted attention. Whilst captive users from the bus and metro group stated being unsatisfied with travel and waiting times and comfort conditions when using the bus. They also have become more concerned about safety overtime when using the bus. These differences in perception are likely a reflection of longer travel times and the negative effects of transfers on satisfaction as shown by Grisé and El-Geneidy (2019). Captive users from the bus and metro mode group of captive riders were mostly unsatisfied with metro services. Even though metro users tend to have higher satisfaction rates than bus users, captive users from both groups reported that they do not feel comfortable on the metro and do not believe that the cost of the trip is reasonable at both points in time.

### Captive-by-choice riders

From before to after the pandemic, the share of captive-by-choice users has decreased (2019 = 27%, 2022 = 23%, -4%). This trend is found across all mode groups (i.e., bus only, metro only, and bus and metro users) as shown in Table 4. Captive-by-choice riders tend to share similar behavioural patterns in terms of telecommuting and frequency of transit use with captive riders. In 2019, 63% of the group frequently used transit to commute to work becoming 47% in 2022. Similarly, 80% did not telecommute in 2019 while 51% did not do so in 2022.

Most captive-by-choice riders believe that they can comfortably take transit to reach their desired destinations (93%) and would recommend transit to family and friends (87%). Except for the bus only group, captive-by-choice users reported higher satisfaction levels than captive riders in relation to bus and metro services. Nonetheless, satisfaction still differ among the analyzed mode groups. At both points in time, the bus only group is mostly unsatisfied with bus operations, including waiting times, travel times and comfort. Even though mostly satisfied, satisfaction levels within the metro only group significantly decreased overtime. These trends lead to a possible continuous decrease in the share of captive-by-choice riders. Whilst bus and metro users maintained their levels of satisfaction towards bus and metro services stable overtime.

**Table 6:** Summary of changes in the transit market between pre- and post-pandemic scenarios by segment

Segment	Captive Riders	Captive by Choice Riders	Choice Riders
*Bus only*	The share of captive bus riders has increased (+ 6%). Telecommuting rates and the number of transit trips to work have remained stable. Captive riders have remained satisfied with bus service.	The share of captive by choice riders has slightly decreased (-2%). Telecommuting rates and the number of transit trips to work have remained stable. Even though satisfaction with bus services has significantly increased among this group, they remain dissatisfied with bus service (e.g., travel and waiting times, comfort, and cost).	The share of choice riders has decreased (-4%). Telecommuting rates have significantly increased from 0 to 2 days a week. The number of trips to work has decreased. At the mean level, no significant changes were found in satisfaction levels. However, the share of unsatisfied riders has increased among choice riders (2019 = 17%, 2022 = 38%). Over time, unsatisfied riders have remained discontent with travel and waiting times and comfort. Those who started telecommuting also report safety concerns.
*Metro only*	The share of captive metro riders has decreased (-4%). Telecommuting rates have significantly increased from 0 to 2 days a week. The number of trips to work has decreased. At the mean level, satisfaction level with metro services has slightly decreased. Their main concern is comfort.	The share of captive by choice riders has decreased (-6%). Telecommuting rates have significantly increased from 0 to 1 day a week. The number of trips to work has decreased. Satisfaction with metro services has decreased at the mean level. Main concerns are costs and waiting times.	The share of choice riders has increased (+ 10%). Telecommuting rates have significantly increased from 0 to 2 days a week. The number of trips to work has decreased. Satisfaction levels with metro services have increased at the mean level. Those unsatisfied have remained discontent with comfort levels while are now also concerned with cost and safety conditions.
*Bus and metro*	The share of captive bus riders has increased (+ 7%). Telecommuting rates have significantly increased from 0 to 1 day a week. The number of trips to work has decreased. At the mean level, satisfaction with bus service has not changed while with metro services has increased. Even so, captive riders tend to be unsatisfied with transit. Their main concerns are travel and waiting times and comfort.	The share of captive by choice riders has decreased (-3%). Telecommuting rates have significantly increased from 0 to 1 day a week. However, no significant mean differences are found in the number of transit trips to work per week. At the mean level, satisfaction with bus service has not changed while with metro services has increased. Captive by choice riders tend to be satisfied with transit services.	The share of choice riders has decreased (-4%). Telecommuting rates have significantly increased from 0 to 2 days a week. The number of trips to work has decreased. At the mean level, satisfaction with both bus and metro services has increased. As expected, satisfaction with metro service is higher than with bus service. There are groups concerned with transit costs and safety at both points in time.
*Overall*	The share of captive riders has slightly increased (+ 2%). Telecommuting rates have significantly increased from 0 to 1 day a week. However, no significant mean differences are found in the number of transit trips to work per week. At the mean level, satisfaction with both bus and metro services has increased over time. Even so, captive riders tend to be unsatisfied with transit services especially when compared to captive by choice and choice riders.	The share of captive by choice riders has decreased (-4%). Telecommuting rates have significantly increased from 0 to 1 day a week. The number of trips to work has decreased. At the mean level, satisfaction with bus services has slightly increased while satisfaction with metro services has remained stable. Captive by choice riders tend to display the highest level of satisfaction compared to choice and captive riders.	The share of choice riders has slightly increased (+ 2%). Telecommuting rates have significantly increased from 0 to 2 days a week. The number of trips to work has decreased. At the mean level, satisfaction with bus services has remained stable while satisfaction with metro services has increased. For bus services, choice riders tend to have satisfaction levels akin to captive riders while, for metro services, their satisfaction levels are akin to captive by choice riders.

### Choice riders

Choice riders are the largest group in the transit market. Their share has slightly increased from 2019 to 2022 (2019 = 57%, 2022 = 59%, + 2%). The metro only group was the only one to increase its share of choice riders (+ 10%) while bus only and bus and metro groups experienced the same reduction rates (-4%). For the two former groups, the reduction in share likely indicates people leaving the market. Choice riders are more prone to be telecommuters than captive or captive-by choice riders. In 2022, 71% of the group telecommuted at least once a week, which reflects an increase of 48% from 2019. Similar shares are found across the three mode groups. On the same note, most choice riders are now infrequent transit riders. In 2019, 46% of the group was composed of infrequent riders which increased to 66% in 2022.

Even though there are clear groups of unsatisfied riders within each mode group as described in Table 3, most remaining choice riders are satisfied with transit. Bus only users have kept similar rates of satisfaction from before to after the pandemic. Metro only users have significantly increased satisfaction levels, including with travel times, waiting times, and comfort levels. Similarly, choice users from the bus and metro group have also increased their satisfaction levels towards both bus and metro services overtime. They also tend to have clear groups of riders concerned with travel costs and safety. Overall, satisfaction levels of choice riders tend to be lower than of captive-by-choice riders and more similar to captive riders especially for bus services.

## Discussions and conclusions

This paper focused on understanding how the transit market changed from pre- to post-pandemic conditions at the overall and mode group level (i.e., bus only, metro only, and bus and metro commuters). We analyse data referring to the 2019 and 2022 waves of the Montréal Mobility Survey concerning travel satisfaction, frequency of transit use, and telecommuting rates of respondents who commuted by transit. In the analysis, a combination of factor and k-mean cluster analyses is applied to derive market segments at both points in time from which changes in the market are examined.

**Fig. 4:**
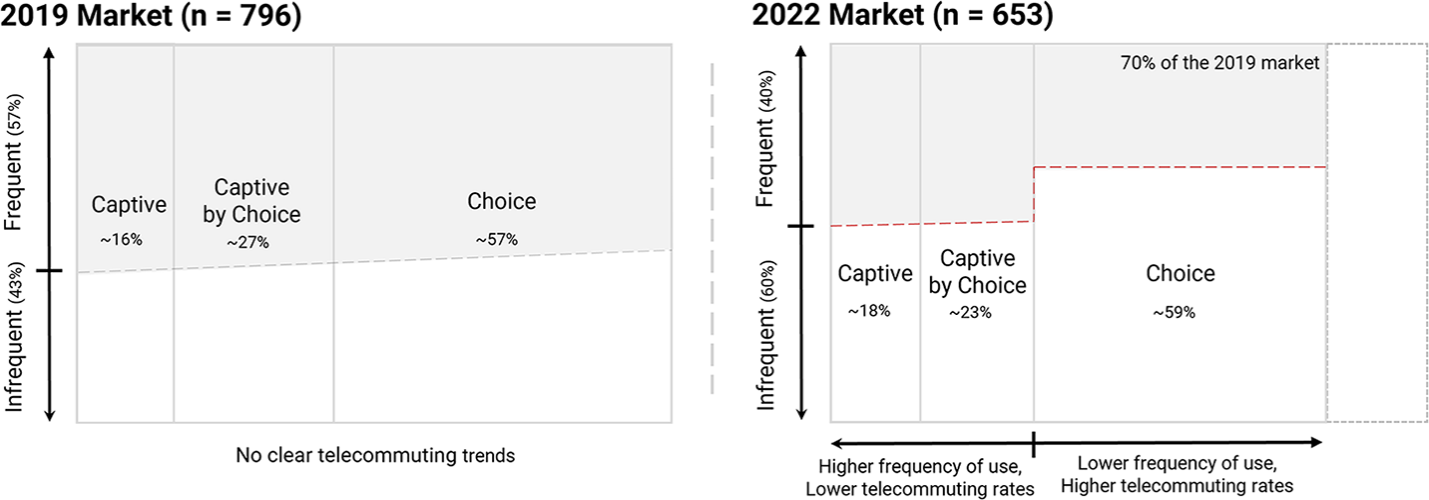
Transit market in pre- (2019) and post-pandemic (2022) conditions

Given relatively small sample sizes, the results reported in this study may under or overrepresent certain groups found in Montréal’s transit market. Even so, the general trends in captive, captive by choice and choice riders in 2019 were consistent with previous research (Van Lierop and El-Geneidy 2017) and we find significant differences in relation to 2022 response patterns. In this sense, while the proportion of captive and choice riders has increased, captive-by-choice riders have shrunk in size. Figure 4 shows an updated market segmentation that builds on previous research (Krizek and El-Geneidy 2007; Van Lierop and El-Geneidy 2017), for simplification the proposed segmentation combines all the studied modes.

In our sample, no new groups have emerged among captive riders across all mode groups. At both points in time, captive riders in the bus only group have remained satisfied while those in the metro only and in the bus and metro groups have continued unsatisfied with transit services. Unsatisfied bus and metro riders tend to have negative perceptions of travel and waiting times and comfort. Therefore, indicating those as potential areas of improvement as highlighted by Mashrur et al. (2023). Overall, captive riders experienced the least reduction in the share of frequent transit riders compared to captive-by-choice and choice riders (2019 = 61%, 2022 = 49%, -13%). Moreover, most remain non-telecommuters (2019 = 84%, 2022 = 52%, -32%). Captive bus users tend to use transit more frequently and to telecommute less than captive metro users.

Within captive-by-choice riders, the metro only group has shifted from a mostly satisfied group to a mostly unsatisfied one, especially in terms of cost and waiting times. On the other hand, the captive-by-choice bus only and bus and metro groups have kept their satisfaction levels stable. Overall, captive-by-choice riders are the most pleased with bus and metro operations and the most willing to recommend transit. These findings reinforce the notion proposed by Van Lierop and El-Geneidy (2017) that captive-by-choice riders become captive to transit because they enjoy their experience and highlight their deeply held commitment to their choice. Thus, indicating loyal tendencies as both factors are connected to loyal behavior (Carvalho et al. 2021; van Lierop and El-Geneidy 2016). Nonetheless, the group faces a trend of reduction in satisfaction levels which could lead to a continuous decrease in the share of captive-by-choice riders. In terms of frequency of use and telecommuting rates, captive-by-choice riders tend to behave similarly to captive riders. Consequently, a high share of the group still uses transit frequently to commute to work (2019 = 63%, 2022 = 47%, -16%) while most do not telecommute (2019 = 80%, 2022 = 51%, -29%).

Choice riders are the ones that changed the most significantly. This group displayed the most increase in telecommuting rates and decrease in frequency of transit use. Clusters of telecommuters are now found across all mode groups which were not present in 2019. Telecommuters are 70%, 66%, and 75% of the bus only, metro only, and bus and metro groups respectively. These sub-markets are also now predominantly composed of infrequent transit users. At the overall level, the share of people who telecommuted at least once a week increased by 48% from 2019 to 2022 (2019 = 23%, 2022 = 71%) while infrequent users increased by 19% (2019 = 46%, 2022 = 66%).

Telecommuting rates seem to be an influential factor in explaining changes in the transit market (e.g., reduction in the market share) as indicated by our findings. The continued impact of telecommuting on transit ridership will depend on a multitude of factors, including the extent to which “back to the office” policies are accepted and implemented. Telecommuting adoption also varies based on socio-demographic characteristics being more popular among younger populations and high-income individuals (Zhu and Wang 2024). Moreover, telecommuting might have allowed people to move farther from offices reinforcing urban sprawl and making transit an inconvenient option (Liu and Su 2021; Yiu et al. 2023). Nonetheless, it is unlikely that telecommuting rates will return to pre-pandemic levels as the practiced has become normalized. The literature is unclear on whether non-work travel among telecommuters has increased or decreased (Zhu and Wang 2024). Even so, choice riders might have been dissuaded from transit towards car (Soria et al. 2023) or active travel (Angel et al. 2023). In the post-pandemic scenario, we find an inverse relationship between frequency of use and telecommute rates, especially among choice riders, which although a straightforward result is indicated in Fig. 4. Similarly, captive, and captive-by-choice riders, which rely on transit for their commute, telecommute at lower rates.

Our findings indicate fundamental changes in the transit market, especially within choice users. The pandemic has caused significant disruptions in behavioural patterns leading to new habits, including how and where people work which in turn affect transit usage. The post-pandemic transit market has shrunk and become mostly composed of infrequent riders who display higher rates of telecommuting. In this sense, the overall changes in behavioural patterns imply that planning transit for 9-to-5 commuter flows needs to be rethought to reflect a new reality. We need to further the understanding of post-pandemic needs and preferences across multiple trip purposes so that transit can better serve the population and, consequently, aid transit ridership recovery. Future research could replicate the analyses done in this paper across multiple cities to examine differences in the patterns of change. Moreover, given data available, studies considering not only work trips, but multiple trip purposes would add to the literature. By doing so, researchers could understand the transit market more holistically and whether certain types of trip purposes have become more popular over time, which could have policy implications. Future studies could also follow the market to understand the impacts of telecommuting and how they change over time.

### Policy recommendations

The findings of this paper indicate that telecommuting is a major factor influencing transit ridership and its recovery. For instance, choice riders have started to telecommute, on average, two days a week leading the share of infrequent or “occasional” users to increase. Therefore, transit agencies will need to adapt as pockets of the market move away from 9-to-5 commute patterns. Palm et al. (2022) reports that regions with higher accessibility by transit and that perceive transit services as having high quality were less affected by reductions in transit use. This highlights the need for agencies to keep improving service provision to retain and encourage ridership recovery and growth. We report policy directions for captive, captive by choice, and choice riders.

### Captive riders

Although captive riders remained taking transit during and after the pandemic, they are mostly doing so for a lack of other options. They were the most affected by service cuts and faced challenges accessing essential services during pandemic times. They report the lowest levels of satisfaction to bus and metro services compared to captive by choice and choice riders, thus reflecting a need to improve their experience to not lose their ridership in the future. Transit agencies need to understand to what extent captive riders, which tend to be low-income and/or people of color, are underserved by transit. In terms of satisfaction, improvements in travel and waiting times and comfort levels are required.

### Captive by choice riders

The share of captive-by-choice riders seems to have decreased. This likely indicates that the number of households and/or people who have chosen alternative travel modes (e.g., car and active travel) to commute, which lead to new habits and behaviors perduring even after the pandemic. Overall, captive by choice riders remain the most satisfied with transit and are willing to recommend transit to friends and family. It is important to cater to their needs as this group tends to be the most loyal to transit although they have the ability to switch to other modes. There are pockets of dissatisfied users, especially among those who take only the bus or only the metro for their commute. Their main concerns are related to travel and waiting times and comfort (same as captive riders) as well as transit costs which should be addressed to retain their ridership.

Broadly, the existence of a captive by choice rider market indicates that there are people willing to not rely on car travel for commute purposes of their own volition, however their market share is significantly smaller than that of choice riders. This may indicate that policies focusing on increasing zero-car households based on a preference for transit has limits, even so increasing transit accessibility and positive perceptions of service may encourage some riders to go carless as transit becomes a more convenient option.

### Choice riders

Choice riders are the largest share of the transit market. They have started telecommuting to a larger extent compared to other groups and, therefore, have started using transit more occasionally. Thus, this group has a substantial influence on transit ridership and recovery. Among unsatisfied choice riders, their concerns resemble those of captive and captive by choice riders. They are discontented with travel and waiting times as well as comfort levels. To a larger extent than in other groups, choice riders are also bothered by transit costs and safety. In this sense, increasing fares would have a negative impact on their perceptions of transit and should be avoided while actions to increase perceived safety should be planned, such as the presence of security guards, security guards, and improved lighting around bus stops and metro stations.
